# Effect of Gender on Development of Hippocampal Subregions From Childhood to Adulthood

**DOI:** 10.3389/fnhum.2020.611057

**Published:** 2020-12-03

**Authors:** Shu Hua Mu, Bin Ke Yuan, Li Hai Tan

**Affiliations:** ^1^School of Psychology, Shenzhen University, Shenzhen, China; ^2^Shenzhen Institute of Neuroscience, Shenzhen, China

**Keywords:** hippocampus, development, MRI, gender, subregion

## Abstract

The hippocampus is known to be comprised of several subfields, but the developmental trajectories of these subfields are under debate. In this study, we analyzed magnetic resonance imaging (MRI) data from a cross-sectional sample (198 healthy Chinese) using an automated segmentation tool to delineate the development of the hippocampal subregions from 6 to 26 years of age. We also examined whether gender and hemispheric differences influence the development of these subregions. For the whole hippocampus, the trajectory of development was observed to be an inverse-u. A significant increase in volume with age was found for most of the subregions, except for the L/R-parasubiculum, L/R-fimbria, and L-HATA. Gender-related differences were also found in the development of most subregions, especially for the hippocampal tail, CA1, molecular layer HP, GC-DG, CA3, and CA4, which showed a consistent increase in females and an early increase followed by a decrease in males. A comparison of the average volumes showed that the right whole hippocampus was significantly larger, along with the R-presubiculum, R-hippocampal-fissure, L/R-CA1, and L/R-molecular layer HP in males in comparison to females. Additionally, the average volume of the right hemisphere was shown to be significantly larger for the hippocampal tail, CA1, molecular layer HP, GC-DG, CA3, and CA4. However, for the presubiculum, parasubiculum, and fimbria, the left side was shown to be larger. In conclusion, the hippocampal subregions appear to develop in various ways from childhood to adulthood, with both gender and hemispheric differences affecting their development.

## Introduction

The hippocampal cortex is an important structure in the limbic system, plays a critical role in memory, and is particularly vulnerable to the effects of aging (Richter-Levin, 2004; Malykhin et al., 2017; Zammit et al., 2017). The hippocampus formation is not a homogeneous structure and is comprised of several subfields with distinctive histological characteristics, including the subiculum (which can be further subdivided into the pre-subiculum, para-subiculum, and the subiculum proper), the four cornu ammonis sectors (CA1–4), and the dentate gyrus (Duvernoy, 2005). These subfields have been shown to play different roles in memory and learning (Yassa and Stark, 2011; Kesner, 2013; Reagh et al., 2014), and subsequently, are affected differently by Alzheimer’s disease (AD) and the normal process of aging (Thal et al., 2000; Mueller et al., 2007; Mak et al., 2017; Wang et al., 2018). Although age-related structural differences in hippocampal subfields have been previously examined (Yassa and Stark, 2011; Krogsrud et al., 2014), the segmentation was not fine-grained. Recently, a computational method for segmenting hippocampal subfields was presented (Iglesias et al., 2015), allowing for a finer differentiation. The atlas can be used to automatically segment the hippocampal subregions in structural magnetic resonance imaging (MRI) images, using an algorithm that can analyze multimodal data and adapt to variations in MRI contrast due to differences in acquisition hardware or pulse sequences. One study has already utilized this segmentation procedure, however, they did not distinguish between the left and right hemispheres (Tamnes et al., 2018). In this study, we utilized the more detailed segmentation method proposed by Iglesias et al. (2015) and examined the parasubiculum, presubiculum, subiculum, CA1, CA3, CA4, GC-DG, HATA, fimbria, molecular layer HP, fissure, and the tail in both the left and right hippocampus.

Despite the numerous lifespan studies that have focused on the developmental differences in the cerebral cortex (Sowell et al., 2003), little is known about the developmental trajectory of the hippocampal formation across its entire lifespan. Longitudinal investigations regarding hippocampal development have suggested that total hippocampal volume stabilizes in children (approximately at 4 years of age); a consistent and monotonic decline is evident in early adulthood, which shows a more rapid rate of decline in the seventh or eighth decades (Raz et al., 2010; Mattai et al., 2011; Sullivan et al., 2011). Results for the adolescent period have been more variable. Some longitudinal studies have found no significant age-related effects (Mattai et al., 2011; Sullivan et al., 2011). Numerous studies have observed volume increases (Dennison et al., 2013), decreases (Tamnes et al., 2013), or a quadratic inverted U-shaped trajectory (Wierenga et al., 2014; Narvacan et al., 2017; Herting et al., 2018). Thus, further examinations of the volume differences of the hippocampus at different ages in a cohesive sample from childhood to adulthood are needed.

Sexual dimorphism in brain development has been a focus of research for quite some time. With the explosion of neuroimaging research, adult male brains have been observed to be approximately 14% larger than female brains (Lenroot et al., 2007). However, sexual dimorphism in the hippocampus has generated a lot of controversies. Most reviews regarding gender-related differences in the human brain have stated that the hippocampus is larger in females than in males (Cahill, 2006; Hines, 2010). Some studies suggest that the hippocampus is larger in females, however, only during childhood (Cosgrove et al., 2007) or adolescence (Neufang et al., 2009). In contrast, other studies have noted greater hippocampal growth in males during adolescence (Suzuki et al., 2005; Bramen et al., 2011; Tamnes et al., 2018). Additionally, several studies support that the human hippocampus is not sexually-dimorphic (Tan et al., 2016). It is important to note that one factor that may be able to reconcile these published differences is age.

Hippocampal asymmetry and laterality have been observed in normal aging, with most reporting a right-greater-than-left asymmetry (Maller et al., 2007; Schmidt et al., 2018). From a molecular, structural, developmental, and sensitivity point of view, the left and the right hippocampus are completely unlike each other (Samara et al., 2011). However, there have been some discrepancies in the literature over the nature of this putative hippocampal asymmetry. Some studies suggest that the observed asymmetry is partly due to a visual perception bias if the volumes are manually traced (Maltbie et al., 2012; Rogers et al., 2012). Also, the subjects of this research mainly focus on adults and the elderly, but lack reports on adolescents. Therefore, in this study we analyzed a large cross-sectional sample comprised of 198 participants, 6–26 years of age, using a novel automated hippocampal segmentation tool. First, we characterize the developmental trajectory of the hippocampal subfields from childhood to adulthood. Second, we identify the differences in the developmental trajectories of the hippocampal subfields in the left and right hemispheres. Third, we investigate whether underlying gender-related differences are present in the volume of the hippocampal subfields from childhood to adulthood.

## Materials and Methods

### Subjects

**Figure 1:**
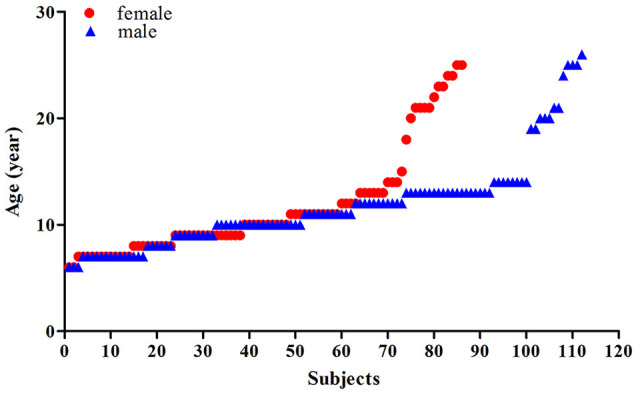
Scatterplots of the ages of the females (red) and males (blue).

This study included two datasets, comprised of a total of 198 healthy Chinese: (1) the first dataset included 89 Chinese participants aged 6–26 years (with an average age of 12.27 ± 6.46 years, 40 females, 49 males) that had been scanned at the Beijing MRI Center for Brain Research of the Chinese Academy; and (2) the second is a public dataset comprised of 109 Chinese participants (Typically-Developing Controls^1^) aged 8–15 years (with an average age of 11.15 ± 2.02 years, 46 females, 63 males) obtained from the Beijing site of the ADHD-200 dataset *via* the International Neuroimaging Data-sharing Initiative (Consortium, 2012). Of the total 198 participants, there were 86 females (with an average age of 11.57 ± 4.98) and 112 males (with an average age of 11.71 ± 4.31), the scatterplots of the ages are shown in Figure 1 and Supplementary Figure 1. For dataset 1, informed consent was obtained from the subjects and/or their parents as appropriate, and the study was approved by the Institutional Review Board of Beijing MRI Center for Brain Research. In dataset 2, the detailed information of the participants was accessed from the public data-sharing website of the ADHD-200 project^1^.

### MRI Acquisition

For dataset 1, all of the MRIs were performed at the Beijing MRI Center for Brain Research of the Chinese Academy of Sciences using a 3 Tesla imager (Siemens, Erlangen, Germany) with a standard head coil. Three-dimensional, high-resolution anatomical scans were acquired using an MPRAGE sequence with the following parameters: TR = 2,300 ms, TE = 3.01 ms, TI = 1,000 ms, FA = 9°; 176 coronal T1-weighted slices with a acquisition matrix = 256 × 256, FOV = 256 × 240 mm^2^ and voxel sizes = 1 × 1 × 1 mm. In dataset 2, the images were acquired using a 3T Siemens Trio scanner with the following scanning parameters: T1-weighted magnetization-prepared rapid acquisition gradient-echo sequences, TR = 2,530 ms, TE = 3.39 ms, TI = 1,100 ms, FA = 7°, acquisition matrix = 256 × 256, FOV = 256 × 256 mm^2^, 128 slices, slice thickness = 1.33 mm, and average = 1.

### Image Processing

Automated segmentation of the hippocampal subfields was performed using the hippo-subfields module in FreeSurfer 6.0, a well-validated open-source software suite that is freely available^2^. The technical details of this automated processing and the specific processing steps are described in detail elsewhere (Iglesias et al., 2015; Tamnes et al., 2018). The reported data were produced using the FreeSurfer default processing stream (recon-all), which includes transformation to Talairach space, intensity normalization for correction of magnetic field inhomogeneities, and removal of non-brain tissues (i.e., skull-stripping). We visually inspected the resulting surface models for motion artifact and manually edited the surfaces in cases where there was overinclusion of the skull, pial matter, or white matter. The hippocampus of each subject was segmented into 12 subfields for each hemisphere: parasubiculum, presubiculum, subiculum, CA1, CA3, CA4, GC-DG, HATA, fimbria, molecular layer HP, fissure, and the tail. The volume of each subfield and the ICV (Intra Cranial Vol) of each subject were then computed and extracted.

### Harmonization Procedures

The removal of side effects from the different datasets was carried out using a posteriori harmonization statistical method named ComBat, which had initially been proposed for genomic studies to correct the so-called batch effect, and has been previously applied to image features from MRIs (Fortin et al., 2017, 2018). We used the “combat” R function provided^3^.

### Statistical Analysis

Partial correlation analysis was used to test the influence of age and gender on each hippocampal subfield. First, we ran correlations between age and each subfield volume as well as the total hippocampal volume, controlling for ICV and gender. Then, we grouped each subfield volume into females and males, and ran correlations between age and volume for each group, controlling for ICV. Analysis of covariance (ANCOVA) was performed to analyze differences in the hippocampal volumes in both its entirety (whole) and its subfields between females and males, using age and ICV as covariates. Paired-samples *t*-test and Wilcoxon signed-rank test were used to analyze differences in the volumes of the hippocampal subregions between the left (L) and the right (R) hemispheres. All of the results were corrected using FDR correction in MATLAB. The significance level for all of the results was set at *P* < 0.05.

## Results

### Effect of Age on the Development of Hippocampal Subregions

**Figure 2:**
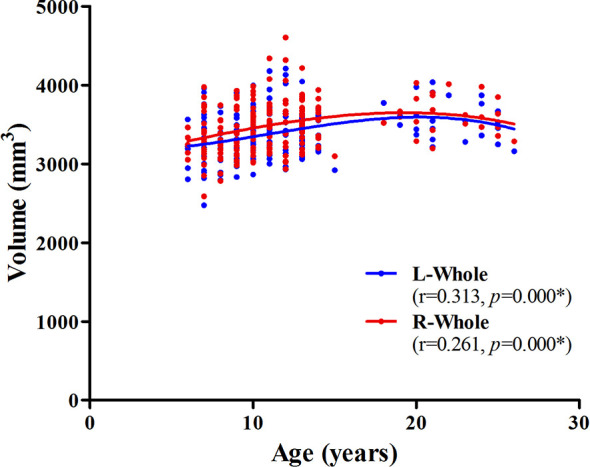
Scatterplots showing whole hippocampal volumes against age, using local smoothing models. Individual left (blue) and right (red) hippocampi are represented by individual lines. Volume is reported in mm^3^ and age is shown in years. L-Whole left whole hippocampus; R-Whole, the right whole hippocampus. *Indicates a significant relationship between volume and age after applying a 5% FDR correction.

**Figure 3:**
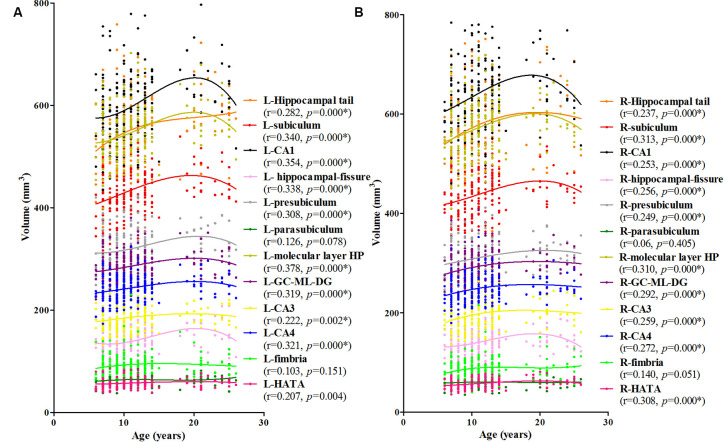
Scatterplots showing the volumes of hippocampal subfields against age, using local smoothing models. Panel **(A)** is the left hippocampus and panel **(B)** is the right hippocampus. Individual hippocampal subregions are represented by individual lines. Volume is reported in mm^3^ and age is shown in years. L-Whole left whole hippocampus; R-Whole, the right whole hippocampus. *Indicates a significant relationship between volume and age after applying a 5% FDR correction.

The association between age and hippocampal volume is illustrated in Figures 2, 3. As shown in Figure 2, positive associations between age and volume are observed bilaterally for the whole hippocampus from 6 to 26 years of age (*P* < 0.001 for both left and right). For both of these volumes, the trajectory of their development was observed to be an inverse-u, in which the volume was shown to increase early, followed by slight decreases (Figure 2). Among these studies of the hippocampal subregions, our results showed significant corrected (*P* = 0.002 for L-CA3, *P* < 0.001 for the others) age-related volume increases for all of the subregions, except the L/R-parasubiculum, L/R-fimbria, and L-HATA (Figure 3). For the L/R-CA1, L/R-molecular layer HP, L/R-subiculum, L-presubiculum, and L/R-hippocampal-fissure, the developmental trajectories showed early increases, followed by sharp decreases. For the L-hippocampal tail, its development followed a consistent increase. For the R-hippocampal tail, R-presubiculum, L/R-GC-DG, L/R-CA3, L/R-CA4, and R-HATA, their development all showed early increases, followed by decelerating decreases.

### Effect of Gender on the Development of the Hippocampal Subregions

#### Left

**Figure 4:**
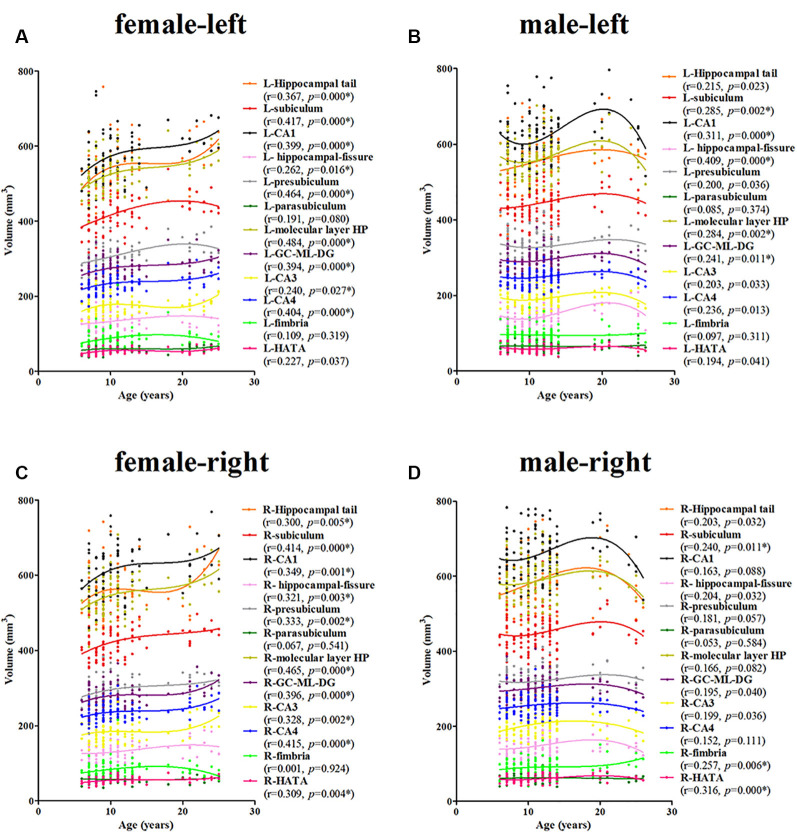
Scatterplots showing the volumes of the hippocampal subfields against age in females and males, using local smoothing models. Panel **(A)** is the left hippocampus in females, panel **(B)** is the left hippocampus in males, panel **(C)** is the right hippocampus in females and panel **(D)** is the right hippocampus in males. Individual hippocampal subregions are represented by individual lines. Volume is reported in mm^3^ and age is shown in years. L, left; R, right. *Indicates a significant relationship of volume with age after applying a 5% FDR correction.

For the females, a significant age-related volume increase was observed for the hippocampal tail, subiculum, CA1, hippocampal-fissure, presubiculum, molecular layer HP, GC-DG, CA3, and CA4 in the left hippocampus (9/12; Figure 4A). For the males, a significant age-related volume increase was found only in the subiculum, CA1, hippocampal-fissure, molecular layer HP, and GC-DG (5/12; Figure 4B). Interestingly, the developmental trajectories of the hippocampal tail, CA1, molecular layer HP, GC-DG, CA3, and CA4 were different between females and males; in females, there is a consistent increase whereas, in males, an early increase is observed followed by a decrease.

#### Right

For the females, a significant age-related increase in the volume of the hippocampal tail, subiculum, CA1, hippocampal-fissure, presubiculum, molecular layer HP, GC-DG, CA3, CA4, and the HATA in the right hippocampus was observed (10/12; Figure 4C). For the males, a significant age-related volume increase was found only in the subiculum, fimbria, and the HATA (3/12; Figure 4D). The developmental trajectories were similar for the left subfields, except for the R-fimbria, which showed a consistent increase in males.

### Effect of Hemisphere and Gender on Average Hippocampal Subregions Volume

#### Hemisphere

**Figure 5:**
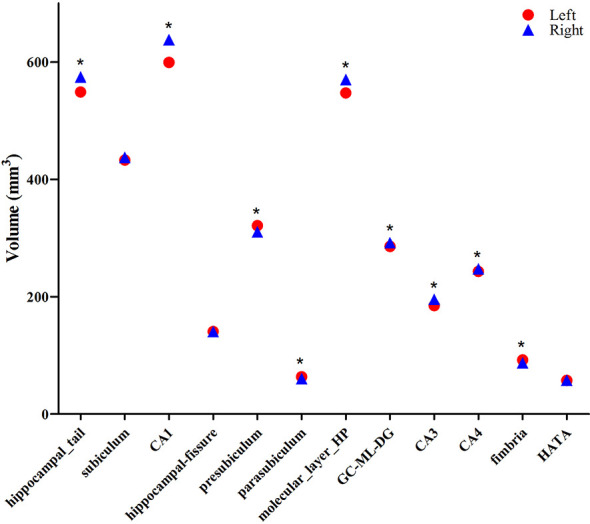
Comparison of the volumes of hippocampal subregions between the left (red circle) and right (blue triangle) hemispheres. *Indicates significant differences between left and right subfields after applying a 5% FDR correction.

The averaged volumes of hippocampal subregions were compared between the left and right hemispheres. For the hippocampal tail, CA1, molecular layer HP, GC-DG, CA3, and CA4, the paired-samples *T*-Test indicated a significant difference between right and left hippocampal volume (*P* < 0.001), with the right hippocampal volume being larger than the left. Inversely, for presubiculum, parasubiculum, and fimbria, the left hippocampal volume was larger than the right, which showed a significant difference (*P* < 0.001). No difference was found for subiculum, hippocampal-fissure and HATA compared between left and right hemispheric volumes (Figure 5).

#### Gender

**Figure 6:**
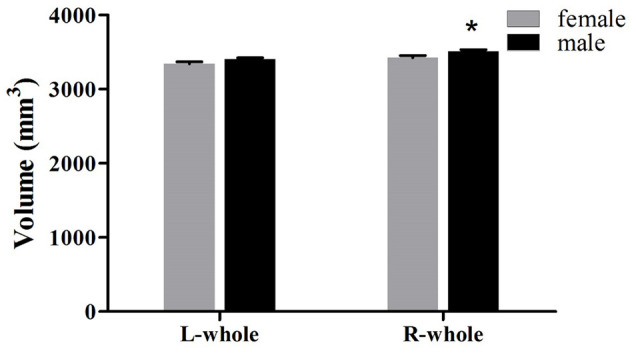
Comparison of whole hippocampal volumes (left and right) between females (gray) and males (black). L, left; R, right. *Indicates significant differences between females and males uncorrected (*P* < 0.05).

**Figure 7:**
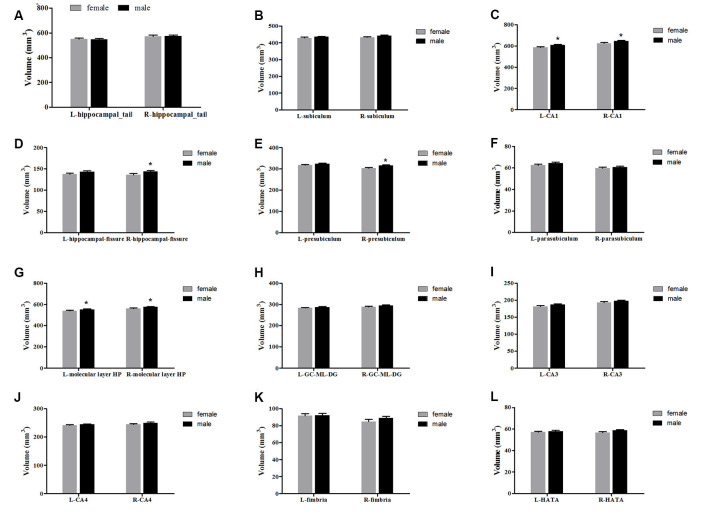
Comparison of the volumes of hippocampal subregions (left and right) between females (gray) and males (black). Panels **(A–L)** showed hippocampal_tail, subiculum, CA1, hippocampal-fissure, presubiculum, parasubiculum, molecular layer HP, GC-ML-DG, CA3, CA4, fimbria and HATA, respectively. L, left; R, right. *Indicates significant differences between females and males uncorrected (*P* < 0.05).

The averaged hippocampal volumes were compared between females and males. The right hippocampus was significantly greater in males in comparison to females (*P* = 0.048; Figure 6). For averaged volumes of hippocampal subregions, a significantly greater volume was observed for the R-presubiculum (*P* = 0.016), L/R-CA1 (*P* = 0.024/*P* = 0.033), L/R-molecular layer HP (*P* = 0.035/*P* = 0.042), and the R-hippocampal-fissure (*P* = 0.044) in males in comparison to females (Figure 7). However, these differences were not significant following a 5% FDR correction.

## Discussion

Three important findings can be drawn from the results of this study: (1) For the whole hippocampus, the development of both the left and right hemispheres followed an inverted U-shaped trajectory from 6 to 26 years of age, however, the hippocampal subregions showed heterogeneous developmental patterns, with significant age-related volume increases for all subregions except for the L/R-parasubiculum, L/R-fimbria, and L-HATA; (2) the development of the hippocampal subregions showed sexual dimorphism from 6 to 26 years of age, showing a consistent increase in females and an early increase followed by a decrease in males for most subregions; and (3) the average volume of the hippocampal subregions between 6–26 years of age showed both gender and hemisphere related differences.

Many studies have investigated the developmental trajectory of the hippocampus from childhood to adolescence. In our present study, the whole hippocampal volume was shown to increase in late childhood and early adolescence, followed by a slight decrease in late adolescence and young adulthood, in agreement with the accumulating evidence from other studies (Wierenga et al., 2014; Coupe et al., 2017; Narvacan et al., 2017; Herting et al., 2018; Tamnes et al., 2018). However, quite a few reported studies on the development of the hippocampal subfields reported different trajectories (Krogsrud et al., 2014; Voineskos et al., 2015; Daugherty et al., 2016; Tamnes et al., 2018). Our results show significant age-related volume increases for the L/R-presubiculum, L/R-subiculum, L/R-CA1, L/R-CA3, L/R-CA4, L/R-GC-DG, R-HATA, L/R-molecular layer HP, L/R-fissure, and L/R-tail, which is in partial agreement with the previously reported studies. Krogsrud et al. (2014) reported that their models for the development of CA1, CA2/3, CA4/DG, presubiculum, and the subiculum estimated a gradually decelerating volume increase until 13–15 years of age, followed by little age-related changes; Lee et al. (2014) also showed that significant age-related increases in the subfield volumes were observed into early adolescence for the right CA3/DG and CA1. However, for the subiculum and fimbria, our results appear to be different from these two studies (Krogsrud et al., 2014; Lee et al., 2014). Also, Tamnes et al. (2018) used the same segmentation method that we used in this study, showing that nonlinear developmental trajectories, with early volume increases, were observed for the subiculum, CA1, and molecular layer; in contrast, the parasubiculum, presubiculum, CA2/3, CA4, and GC-DG showed linear volume decreases from 8 to 26 years of age. Unfortunately, they did not distinguish between the left and right hippocampal subregions. Thus, the differences between our results may be largely due to the variations in the hippocampal segmentation methods. Additionally, this raises the question of whether the developmental differences in the hippocampal subregions are constrained by differing developmental trajectories for the left vs. the right hippocampus.

Although the developmental trajectories for the left and right hippocampus are similar for all of the subregions, except for HATA, the average volume (between 6 and 26 years of age) for most of the hippocampal subregions are different. For 6/12 subregions (hippocampal tail, CA1, molecular layer HP, GC-DG, CA3, and CA4), the right hippocampal volume is larger than the left; for the other 3/12 (presubiculum, parasubiculum, and fimbria), a reverse pattern was identified. Although there are no strong reasons to predict hemispheric differences in hippocampal development, our results are consistent with previous studies, indicating more robust developmental differences in the right compared to the left hippocampus (Gogtay et al., 2006; DeMaster et al., 2014; Lee et al., 2014). DeMaster et al. (2014) found that adults had a smaller right hippocampal head, larger hippocampal body bilaterally, and smaller right hippocampal tail in comparison to children. Gogtay et al. (2006) reported more delayed developmental changes in the right, as compared to the left, hippocampus. Similarly, the right hippocampus was not larger than the left at the baseline, but increased more than the left during this developmental period, resulting in it being significantly larger at the later time-point (Dennison et al., 2013). Furthermore, asymmetries in the nature and extent of associations have been previously reported, with the right hippocampus being more likely to exhibit significant associations with a host of factors, including gender differences during puberty (Bramen et al., 2011). This study is the first to detect hemispheric differences in 12 hippocampal subregions between childhood and adulthood.

When the samples were separated by gender, the developmental trajectory for each subfield suggests some qualitative differences compared to the entire sample. Especially for the hippocampal tail, CA1, molecular layer HP, GC-DG, CA3, and CA4, in which a consistent increase is observed for females and an early increase followed by a decrease in males. Gender-related differences in the trajectory of hippocampal development is still a hotly debated topic. When estimating volume changes based on both age and pubertal development, the hippocampus increases in volume over puberty (7–20 years of age) in both females and males (Goddings et al., 2014). Similarly, several longitudinal studies have not found gender-related differences in developmental trajectories (Dennison et al., 2013; Wierenga et al., 2014; Tamnes et al., 2018). However, another study published by Neufang et al. (2009), which examined correlations between testosterone and brain volumes in boys and girls (8–15 years of age), reported that postpubertal males have significantly reduced hippocampal volumes compared to postpubertal females. Our present results are also consistent with this study, showing a decrease in volume after puberty in males in most hippocampal subregions. In addition to the above two views, a Meta-analysis revealed that the human hippocampus is not sexually-dimorphic. Meta-regression revealed no effect of age on the gender differences in the left, right, or bilateral hippocampus volumes (HCV; Tan et al., 2016). However, they did find that human males of all ages exhibit a larger HCV than females, but adjusting for individual differences in TBV or ICV results in no reliable gender-related differences. In the present study, we also adjusted the ICV in the statistical models, and the gender differences in the averaged hippocampal volumes are found for the right whole hippocampus, R-presubiculum, R-hippocampal-fissure, L/R-CA1, and L/R-molecular layer HP, which are greater in males as compared to females. Using the same segmentation method, Tamnes et al. (2018) also reported that boys had larger volumes than girls for all hippocampal subregions, except for the hippocampal fissure. Unlike us, they did not distinguish between the left and right hippocampus. Because of the hemispheric effect on hippocampal development, it is necessary to compare them separately. Regrettably, there is a gap in our data between 15–20 years old and the young adults might be under-represented. The “inverse-u” relationship observed in males but not in females might be affected by the uneven representations of the data points.

In conclusion, our results indicate that hippocampal subfields develop in various ways across childhood, adolescence, and early adult (6–26 years of age) with nonlinear trajectories, except for the L/R-parasubiculum, L/R-fimbria, and L-HATA. Additionally, gender differences in the development of most subregions were observed, especially for the hippocampal tail, CA1, molecular layer HP, GC-DG, CA3, and CA4, with a consistent increase in females and an early increase followed by a decrease in males. The averaged hippocampal volumes in the right whole hippocampus, R-presubiculum, R-hippocampal-fissure, L/R-CA1, and L/R-molecular layer HP are observed to be larger in males as compared to females. Finally, we also found that hemisphere differences exist in most hippocampal subregions, except for the subiculum, hippocampal-fissure, and HATA. In summary, our study is the first to examine the effects of age, sex, and hemisphere differences in the 24 hippocampal subfields from childhood to adulthood. Further studies are needed to confirm our findings and determine the value of these measurements for the differentiation between normal aging vs. other diseases, which affect the hippocampal subfields.

## Data Availability Statement

The raw data supporting the conclusions of this article will be made available by the authors, without undue reservation.

## Ethics Statement

The studies involving human participants were reviewed and approved by Institutional Review Board of Beijing MRI Center for Brain Research. Written informed consent to participate in this study was provided by the participants, and/or where necessary, the participants’ legal guardian/next of kin.

## Author Contributions

LT and SM designed this study and revised and guided the experiment. SM wrote this manuscript and participated in the whole experiment process. BY and SM managed the whole experiment and analyzed the data. All authors contributed to the article and approved the submitted version.

## Conflict of Interest

The authors declare that the research was conducted in the absence of any commercial or financial relationships that could be construed as a potential conflict of interest.

## Footnotes

^1^http://fcon_1000.projects.nitrc.org/indi/adhd200/

^2^http://surfer.nmr.mgh.harvard.edu/

^3^https://github.com/Jfortin1/ComBatHarmonization
