# Supplementary material for: Effect of Gender on Development of Hippocampal Subregions From Childhood to Adulthood
Source: Front Hum Neurosci. 2020 Dec 3;14:611057. doi: 10.3389/fnhum.2020.611057 (PMC7744655; doi:10.3389/fnhum.2020.611057)
Supplement: Supplementary file 1 [file Table_1.DOCX]

Supplementary Figure 1. Scatterplots of the ages of the females (red) and males (blue). Database 1 and database 2 are presented separately.
