# Supplementary figures and images for: Effect of Gender on Development of Hippocampal Subregions From Childhood to Adulthood
Source: Front Hum Neurosci. 2020 Dec 3;14:611057. doi: 10.3389/fnhum.2020.611057 (PMC7744655; doi:10.3389/fnhum.2020.611057)

Supplementary Figure 1

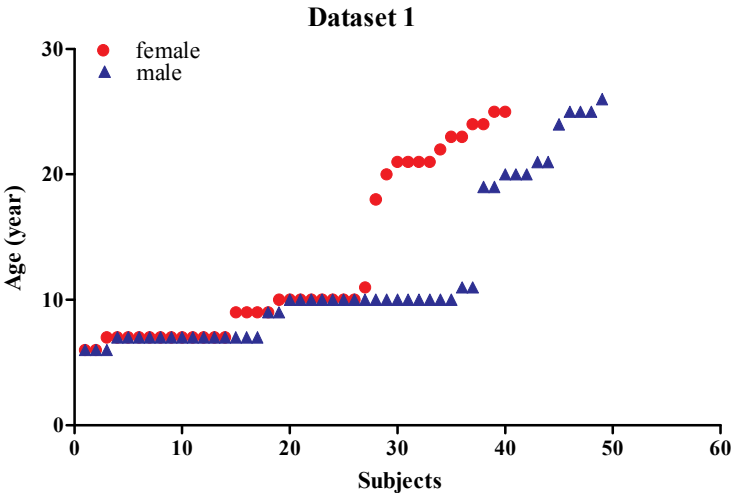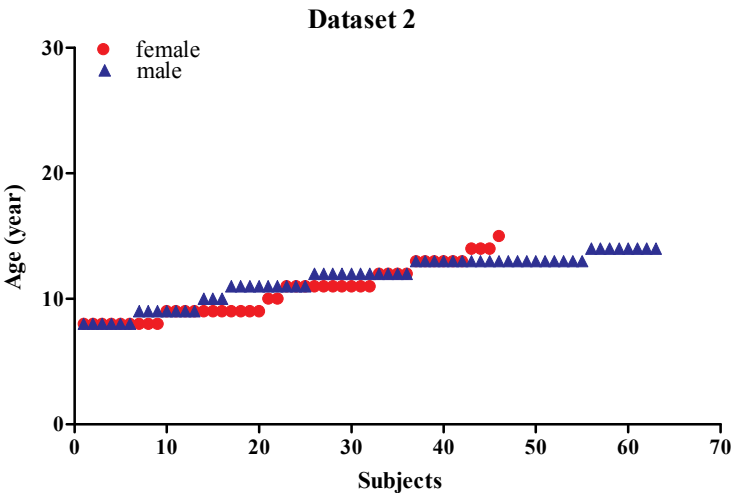

Supplement: Supplementary file 2 [file Data_Sheet_1.PDF]
